# No robust reduction of infarct size and no-reflow by metoprolol pretreatment in adult Göttingen minipigs

**DOI:** 10.1007/s00395-023-00993-4

**Published:** 2023-06-08

**Authors:** Petra Kleinbongard, Helmut Raphael Lieder, Andreas Skyschally, Gerd Heusch

**Affiliations:** Institute for Pathophysiology, West German Heart and Vascular Center, University of Essen Medical School, University of Duisburg-Essen, Hufelandstr. 55, 45147 Essen, Germany

**Keywords:** Cardioprotection, Coronary microvascular obstruction, Infarct size, Metoprolol, Myocardial infarction, Reperfusion

## Abstract

Whereas prior experiments in juvenile pigs had reported infarct size reduction by intravenous metoprolol early during myocardial ischaemia, two major clinical trials in patients with reperfused acute myocardial infarction were equivocal. We, therefore, went back and tested the translational robustness of infarct size reduction by metoprolol in minipigs. Using a power analysis-based prospective design, we pretreated 20 anaesthetised adult Göttingen minipigs with 1 mg kg^−1^ metoprolol or placebo and subjected them to 60-min coronary occlusion and 180-min reperfusion. Primary endpoint was infarct size (triphenyl tetrazolium chloride staining) as a fraction of area at risk; no-reflow area (thioflavin-S staining) was a secondary endpoint. There was no significant reduction in infarct size (46 ± 8% of area at risk with metoprolol vs. 42 ± 8% with placebo) or area of no-reflow (19 ± 21% of infarct size with metoprolol vs. 15 ± 23% with placebo). However, the inverse relationship between infarct size and ischaemic regional myocardial blood flow was modestly, but significantly shifted downwards with metoprolol, whereas ischaemic blood flow tended to be reduced by metoprolol. With an additional dose of 1 mg kg^−1^ metoprolol after 30-min ischaemia in 4 additional pigs, infarct size was also not reduced (54 ± 9% vs. 46 ± 8% in 3 contemporary placebo, n.s.), and area of no-reflow tended to be increased (59 ± 20% vs. 29 ± 12%, n.s.).

Infarct size reduction by metoprolol in pigs is not robust, and this result reflects the equivocal clinical trials. The lack of infarct size reduction may be the result of opposite effects of reduced infarct size at any given blood flow and reduced blood flow, possibly through unopposed alpha-adrenergic coronary vasoconstriction.

**Supplementary Information:**

The online version contains supplementary material available at 10.1007/s00395-023-00993-4.

## Introduction

The translation from successful preclinical studies on cardioprotective mechanical and pharmacological interventions to the benefit of patients with reperfused acute myocardial infarction has been difficult so far [33]. This difficulty has been largely attributed to the advanced age of patients and their co-morbidities and co-medications, whereas the preclinical studies were usually done in young and healthy animals [17, 30, 47]. However, recently more thorough preclinical studies revealed that some cardioprotective interventions are not really robust and that there may have been a publication bias for positive studies [55]. In fact, not only age [3] or co-morbidities and co-medications [17, 47] may interfere with cardioprotection also in animal experiments, but also a genetic predisposition may be a primordial obstacle to cardioprotection, as recently demonstrated for rats with remote ischaemic conditioning [71] and for Ossabaw minipigs with ischaemic preconditioning [34, 50, 81].

The difficulty of translation also relates to cardioprotection by the beta blocker metoprolol. The laboratory of Ibanez has convincingly demonstrated infarct size reduction in immature Yorkshire and Large White pigs with left anterior descending coronary artery (LAD) occlusion and reperfusion [41, 43, 59]. In their preclinical studies, they emphasised the importance of timing in the administration of metoprolol; metoprolol was only cardioprotective when given before reperfusion, [41] longer exposure was better than shorter exposure during ischaemia, [22] and early treatment with metoprolol delayed the natural progression of infarction by about 10–15 min, [59] Mechanistically, Ibanez and co-workers attributed the cardioprotection by metoprolol to a non-class effect through attenuation of inflammation and improvement of the coronary microcirculation [10, 21, 41].

The encouraging preclinical data were confirmed in the METOCARD-CNIC (Effect of Metoprolol in Cardioprotection During an Acute Myocardial Infarction) trial, in which intravenous metoprolol given to patients early during an ST-segment elevation myocardial infarction (< 6 h after symptom onset and before reperfusion) reduced infarct size on magnetic resonance imaging (MRI) [42]. These patients also had less coronary microvascular obstruction on MRI, [21] better left ventricular (LV) function outcome and less heart failure admission on follow-up [66, 67]. As in the pig experiments, longer exposure to metoprolol was associated with smaller infarct size [22]. Retrospective analysis of sequential ECGs also confirmed the delay of infarct progression, and the ECG analysis correlated to the MRI data on infarct size, coronary microvascular obstruction and LV function [14].

In contrast to the METOCARD-CNIC trial, the larger phase III EARLY-BAMI (Early beta blocker administration before primary percutaneous coronary intervention (PCI) in patients with ST-elevation myocardial infarction trial) trial was neutral with respect to infarct size, as reflected by creatine kinase release and MRI, and clinical outcome, [68, 69] and this discrepancy may relate to more unfavourable timing and dosing of metoprolol in EARLY-BAMI than METOCARD [45, 59].

Apparently, the translation of the encouraging preclinical data on metoprolol to cardioprotection in patients with acute myocardial infarction was equivocal. We now went back to investigate how robust the preclinical data are and for that purpose used a different strain of pigs (Göttingen minipigs rather than Yorkshire or Large White pigs) which were mature rather than immature, and used a different anaesthesia regimen, i.e. one which we also use in cardiosurgical patients in our institution [78]. To follow the advice of Ibanez et al. to use early treatment, [59] we administered metoprolol just before coronary occlusion to give it the best chance to exert cardioprotection, and we gave an even somewhat higher dose. To generate robust data, we also used a power analysis-based prospective study design [4, 77] with a pre-specified separate analysis of female and male pigs [48].

## Methods

The authors declare that all supporting data of the present study are available in the article. Original data underlying this article will be shared on reasonable request to the corresponding author. Unless otherwise specified, materials were obtained from Sigma-Aldrich (Deisenhofen, Germany).

The experimental protocols were approved by the Bioethical Committee of the district of Düsseldorf (G1868/21) and conform to the guidelines from Directive 2010/63/EU of the European Parliament on the protection of animals used for scientific purposes. We followed the ARRIVE 2.0 guidelines [64, 65]. A full description of the intended experimental design and analysis has not been published in a preclinical registry. Experiments in pigs were performed between June 2022 and December 2022.

### Experimental preparation

Validity of animal species and model selection: This in vivo experimental model in pigs replicates aspects of the human reperfused myocardial infarction [39]. Göttingen minipigs (females and males) were purchased from Ellegaard, Dalmose, Denmark. Pigs were fed with standard chow (twice 300 g/day, #V4133. ssniff, Soest, Germany), had access to water ad libitum and were kept in tiled rooms (~ 2 m^2^/pig) with straw-bedding at 12 h/12 h light/dark cycles. There was a daily visual inspection of pigs’ health by animal caretakers and veterinarians. All behavioural abnormalities were recorded and monitored; only inconspicuous, apparently healthy pigs were included in the experiment. Female pigs were 19 ± 2 and male pigs 19 ± 5 months old. Females pigs weighed 46 ± 7 and male pigs weighed 39 ± 9 kg. Pigs were sedated with flunitrazepam (i.m.: 0.4 mg kg^−1^). Anaesthesia was induced with etomidate (i.v.: 0.3 mg kg^−1^, Hypnomidat; Voorschoten, The Netherlands) and sufentanil (i.v.: 1 µg kg^−1^, Sufentanil-hameln, Hameln, Germany). Anaesthesia was maintained with isoflurane (2%, TEVA, Eastbourne, United Kingdom) during artificial ventilation with room air (tidal volume: 8–10 mL kg^−1^, respiratory rate: 10–16 breaths min^−1^, inspiratory peak pressure 18–25 cm H_2_O, positive end-expiratory pressure: 5–7 cm H_2_O). Muscle relaxation during electrosurgery was induced with a single bolus of rocuronium (i.v.: 0.6 mg kg^−1^, B. Braun, Melsungen, Germany). This anaesthetic regimen is identical to that used in our institution for patients undergoing surgical coronary revascularisation. [78] The pigs were placed on a heated table and covered with heated blankets to keep oesophageal temperature between 36.0 and 38.0 °C. ECG-lead II was continuously recorded using a single-channel, calibrated amplifier. A midline cervical incision was performed. The left jugular vein was cannulated for volume replacement and intravenous drug administration, and the right common carotid artery was cannulated to measure arterial pressure. The heart was exposed by a left lateral thoracotomy and instrumented with a micromanometer (DPT-6000, Codan-PVB, Forsting, Germany) in the left ventricle to measure left ventricular pressure (LVP) and a Teflon catheter in the left atrium for the injection of coloured microspheres [53]. The distal aortic arch was cannulated to withdraw the reference sample for regional blood flow measurement. The left anterior descending coronary artery (LAD) was dissected and prepared distal to its second diagonal branch for later coronary occlusion.

### Regional myocardial blood flow

Coloured microspheres were recovered from transmural myocardial samples taken from the central area at risk by digestion with 4 mol L^−1^ KOH and subsequent filtration (8 µm pore size, Pieper Filter, Bad Zwischenahn, Germany). Fluorescent dye was resolved from microspheres and quantified in a spectrophotometer (F-7100, Hitachi High-Tech, Krefeld, Germany). Blood flow was calculated as blood flow per tissue mass.

### Area at risk, infarct size and no-reflow

Thirty ml of warmed 4% thioflavin-S solution (Morphisto, Frankfurt, Germany) was filtered through a 0.2 µm syringe-filter to remove particulate debris and slowly infused into the left atrium to demarcate non-perfused areas of the left ventricle after 180-min reperfusion [7, 74]. Thereafter, the LAD was re-occluded at the same location as for the index ischaemia, and 5 ml blue dye (Patentblau V, Guerbet GmbH, Sulzbach, Germany) was quickly injected into the left atrium to delineate the area at risk as remaining unstained.

The heart was quickly removed from the chest, rinsed with cold saline, and cut into 5 slices perpendicular to the ventricular long axis. The tissue slices were examined under ultraviolet light (340–360 nm, VL-UVA 135.11, Vilber Louramat, Eberhardzell, Germany). Areas without yellow–green fluorescence (thioflavin-S-negative) were encircled by incisions. After documenting the slices using a digital camera, the slice shape, the thioflavin-S-negative areas, and the demarcated area at risk were transferred to a transparent film. Thereafter, infarcted tissue was demarcated by triphenyl tetrazolium chloride (TTC) staining (1% dissolved in 90 mmol L^−1^ sodium phosphate buffer containing 8% dextran, Roth, Karlsruhe, Germany). The TTC-stained slices were again photographed and together with the tissue areas which remained unstained by TTC transferred to the same transparent film which was used to document the area at risk and the no-reflow areas. Particular care was taken to proper re-align the slices using “landmarks”, such as the position of papillary muscles and the incisions surrounding no-reflow areas.

The transparent films were scanned and analysed using digital planimetry. The following areas were calculated and averaged for both sides of each slice: total area of the left ventricle, the area at risk, the area of TTC-negative tissue (infarcted), the area of thioflavin-S-negative tissue within the infarcted tissue (no-reflow); the average areas of both sides were then calculated. The weights of all slices were then summed up, and the total tissue masses for the area at risk, the no-reflow area, and for the infarcted area of a given heart were calculated. In addition, the area at risk was calculated as a fraction of the left ventricle, infarct size was calculated as a fraction of the area at risk, and the area of no-reflow was calculated as a fraction of infarct size and as fraction of area at risk.

### ECG analysis

ST-segment elevation was quantified as previously described [1, 46]. A chest surface ECG was continuously recorded using a single-channel, calibrated (1 mV reference) amplifier. Due to the surgical preparation and use of a metal rib retractor, the recorded ECG-lead appeared similar to a V2 Wilson lead in humans. The ST-segment elevation was defined as the amplitude difference between a point 30 ms before the P-wave and a second point 20 ms after the J-point [1, 11]. The analysis was performed offline using digital calipers (Labchart 8, AD Instruments Pty Ltd, New South Wales, Australia).

ST-segment elevation was quantified at baseline and at 5-min intervals during the 60 min of coronary occlusion and the first 15 min of reperfusion. ST-segment elevation data were excluded from the analysis of ST-segment elevation when the P-wave could not be identified due to poor signal quality, when the voltage amplitude of the R-wave was less than 300 µV or when episodes of ventricular fibrillation and termination by electric countershock during index ischaemia and/or reperfusion did not permit ECG analysis within the respective time frame.

### Protocols

#### *Ischaemia/reperfusion (I/R)*

At baseline, after stabilisation for at least 30 min, systemic haemodynamics and regional myocardial blood flow were measured. As a placebo control, 10 ml saline was infused intravenously. After intravenous infusion of unfractionated heparin (2.500 IU, Heparin-Natrium-ratiopharm, Ulm, Germany) the LAD was occluded distal to its second diagonal branch using a microvascular clamp (TKL-1, BIOVER^R^, Hergiswill, Switzerland). Heparin (2.500 IU) was again given at 25- and 55-min coronary occlusion. At 5- and 55-min coronary occlusion, systemic haemodynamics and regional myocardial blood flow were measured again. Reperfusion was induced after 60-min coronary occlusion by quick removal of the vascular clamp and visually confirmed by the reappearance of red colour on the surface of the reperfused myocardium. Systemic haemodynamics and regional myocardial blood flow were again measured at 10- and 180-min reperfusion. Ventricular fibrillation during ischaemia or reperfusion, as identified from the continuous lead II ECG recording, was immediately terminated by intra-thoracic defibrillation (up to 50 Ws; 6/4 ms biphasic pulse; Zoll R Series Monitor & Defibrillator, Zoll Medical Cooperation, Chelmsford, MA, USA). We did not use any anti-arrhythmic or inotropic agents during resuscitation since they might interfere with the infarction process and/or cardioprotection [13, 40, 75]. At the end of the experiment, pigs were euthanised by intracardiac injection of 20 ml potassium chloride (1 mol L^−1^).

#### *Metoprolol* + *I/R*

The experimental protocol was identical to that of I/R, except that metoprolol (1 mg kg^−1^ i.v. over 10 min in 10 ml saline, Sigma Aldrich, Deisenhofen, Germany) was administered after baseline measurements before ischaemia. The dose of metoprolol was determined with reference to prior studies [41, 59] and to preliminary experiments in our preparation where metoprolol (1 mg kg^−1^ i.v.) attenuated the increments of LV dp dt^−1^_max,_ heart rate and LVP in response to a bolus of 5 μg adrenaline i.v. (Suprarenin, Cheplapharm, Greifswald, Germany). In an exploratory analysis in 4 additional pigs (3 females, 1 male) which were not included in the prospective study design, metoprolol (1 mg kg.^−1^ i.v.) was given before ischaemia and again over 10 min starting at 30-min ischaemia; these pigs were compared to 3 separate contemporary (within less than 3 weeks) female placebo controls; these separate contemporary controls were used to account for potential seasonal differences in infarct size [51] and no-reflow [79].

### Power analysis

We defined infarct size as the primary endpoint, type I error α as 0.05 and type II error 1-β as 0.90. To estimate the required number of experiments we used infarct size data from two publications. In Yorkshire pigs (*N* = 10), metoprolol (3 × 2.5 mg i.v. starting at 15-min ischaemia, *n* = 6) reduced infarct size after 90-min ischaemia and subsequent reperfusion from 94 ± 7% to 68 ± 15% of the area at risk (*n* = 4 placebo) [43]. The calculated effect size d_Cohen_ is 2.1, requiring a total number of 10 experiments (G-Power 3.1; University of Düsseldorf, Germany). In 3-month-old male Large White pigs (N = 116), metoprolol (0.75 mg kg^−1^ i.v. at 20-min ischaemia) reduced infarct size from 92 ± 9% (*n* = 58) to 75 ± 14% of the area at risk (n = 58) when ischaemia durations between 30 and 60 min were pooled [59]. The resulting effect size d_Cohen_ is 1.5, requiring a total number of *N* = 18 experiments. The mean effect size of d_Cohen_ = 1.8 for both these prior studies results in a total of *N* = 14 experiments. To account for differences in pig strain, maturity/age, sex, experimental preparation incl. anaesthesia, and protocol incl. notably the duration of ischaemia and reperfusion between these prior studies and our present one, and to provide a balanced number of experiments without and with metoprolol for both, male and female Göttingen minipigs, respectively, we conservatively increased the total number of experiments to *N* = 20. We used an oesophageal temperature at baseline before ischaemia in the range of 36.0–38.0 °C and a blood flow during ischaemia < 0.1 ml min^−1^ g^−1^ as a-priori inclusion criteria and conducted the study until for each sex in each group at least *n* = 5 experiments with infarct size data were available. In our hands, at blood flows > 0.1 ml min^−1^ g^−1^ there is a possibility that the coronary occlusion clamp may not have been tight, possibly secondary to cardiac movements with fibrillation/defibrillation [48]. Pigs were randomised (using sealed envelopes) to an I/R or metoprolol+I/R protocol, respectively. A pre-specified sub-group analysis for female and male pigs was performed. Also, we analysed the inverse relationship between infarct size and regional myocardial blood flow and its potential shift by metoprolol as a secondary endpoint.

### Data and statistical analysis

The investigator who quantitatively assessed haemodynamics, regional myocardial blood flow, infarct size and area of no-reflow was blinded to the protocol. Explorative statistics were performed on all data to test for normal distribution (Shapiro–Wilk-test) and to identify potential outliers. Data are presented as means ± SD; individual data on infarct size and no-reflow are also presented as scatterplots. Statistical package SAS 9.4 (Cary, NC, USA) was used to analyse haemodynamic, ST-segment elevation, and regional myocardial blood flow data by two-way analysis of variance (protocol, time) for repeated measures. When analysis of variance indicated a significant main effect or interaction, least square means were used for further analysis of simple effects. Data on age, body weight, number of episodes of ventricular fibrillation/defibrillation during ischaemia, area at risk, infarct size and no-reflow were analysed by unpaired two-sided t-tests. The number of episodes of ventricular fibrillation/defibrillation during ischaemia and the no-reflow data were not normally distributed and subjected to an aligned-rank transformation (art) before analysis. In an exploratory analysis, infarct size and no-reflow area were compared between the 4 pigs with twice 1 mg kg^−1^ i.v. metoprolol and the 3 placebo pigs by unpaired two-sided t-tests. The relationships between infarct size and regional myocardial blood flow without and with metoprolol were analysed by linear regression and compared by analysis of covariance. Differences were considered significant at the level of *p* < 0.05.

The data and statistical analysis comply with the recommendations on experimental design and analysis in pharmacology [12].

## Results

One pig which had received metoprolol was lost from intractable ventricular fibrillation during ischaemia. One pig of the metoprolol and one pig of the placebo group were excluded from analysis because blood flow during ischaemia was > 0.1 ml min^−1^ g^−1^.

### Magnitude and duration of beta blockade by metoprolol

The peak haemodynamic responses in response to an intravenous bolus of 5 µg adrenaline were almost completely inhibited 5 min after intravenous infusion of 1 mg kg^−1^ metoprolol; the inhibition was still substantial after 30 min but had somewhat subsided by 60 min (Table 1).

**Table 1:**
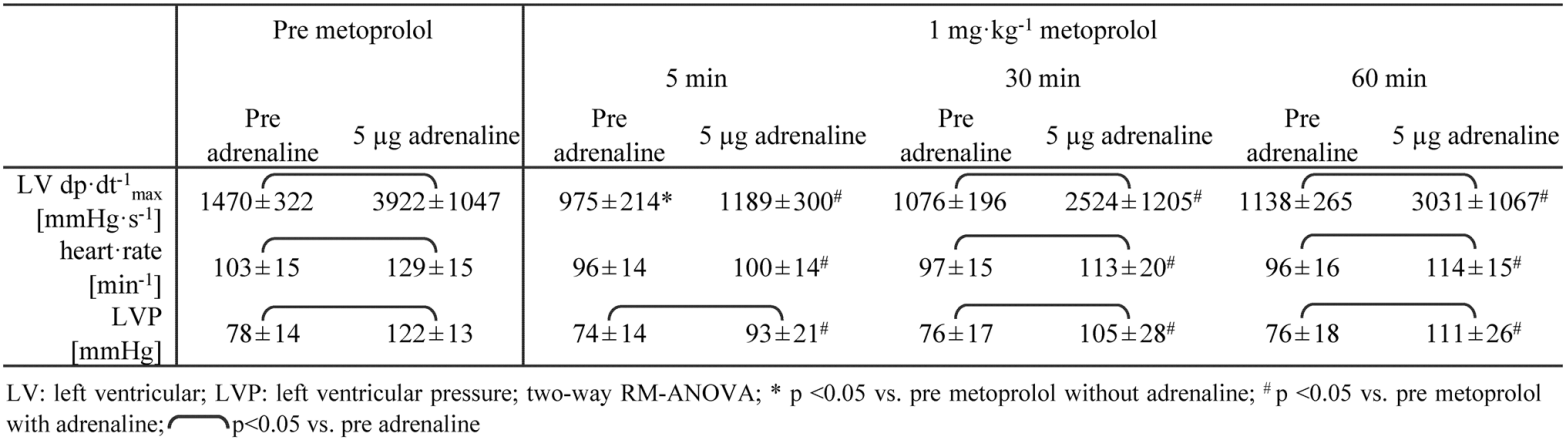
Peak haemodynamic responses to adrenaline after beta blockade with 1 mg kg^−1^ metoprolol

### Haemodynamics

At baseline, heart rate and LVP were not different between the metoprolol and placebo groups. LVP decreased over the duration of ischaemia/reperfusion in pigs without and with metoprolol, and heart rate increased; the increase in heart rate was slightly attenuated with metoprolol. The blood flow reduction during ischaemia was equally severe. Heart rate and LVP in pigs undergoing ischaemia/reperfusion without versus with twice 1 mg kg^−1^ (pretreatment plus treatment from 30- to 40-min ischaemia) metoprolol did also not differ between the groups (see Supplemental Table).

### ECG

ST-segment elevations were not different between the metoprolol and placebo groups, when normalised to their baseline (see Supplemental Figure). The number of episodes with ventricular fibrillation/defibrillation was not different between the metoprolol and placebo groups (Table 2).

**Table 2:**
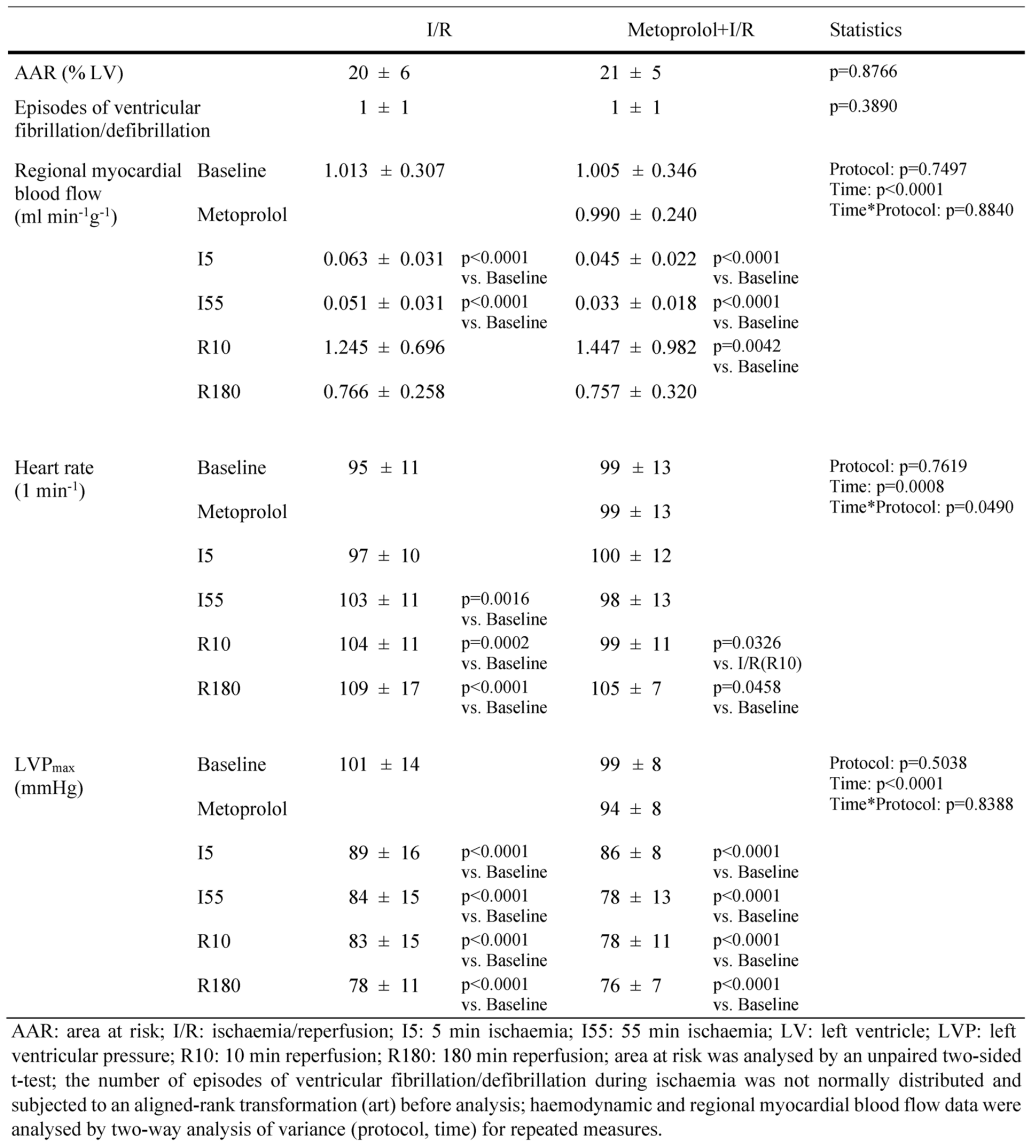
Area at risk, regional myocardial blood flow, heart rate and left ventricular pressure in pigs undergoing ischaemia/reperfusion without and with 1 mg kg^−1^ metoprolol

### Infarct size and no-reflow area

**Fig. 1:**
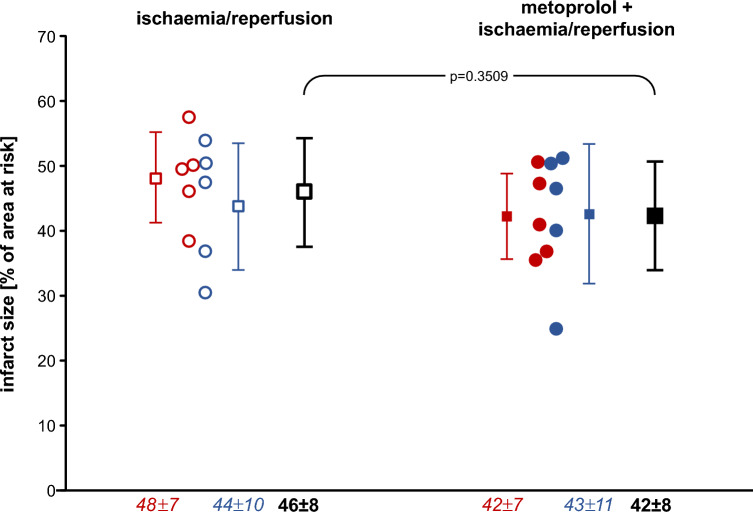
Infarct size following ischaemia/reperfusion without (open symbols) and with 1.0 mg kg^−1^ metoprolol (filled symbols) in female (red) and male (blue) Göttingen minipigs; individual data (circles) and means (squares) with SD; means ± SD were also given as numerical data; females and males with ischaemia/reperfusion (n = 5, each); females and males with metoprolol + ischaemia/reperfusion (*n* = 5, each). Data were analysed by unpaired two-sided t-tests

**Fig. 2:**
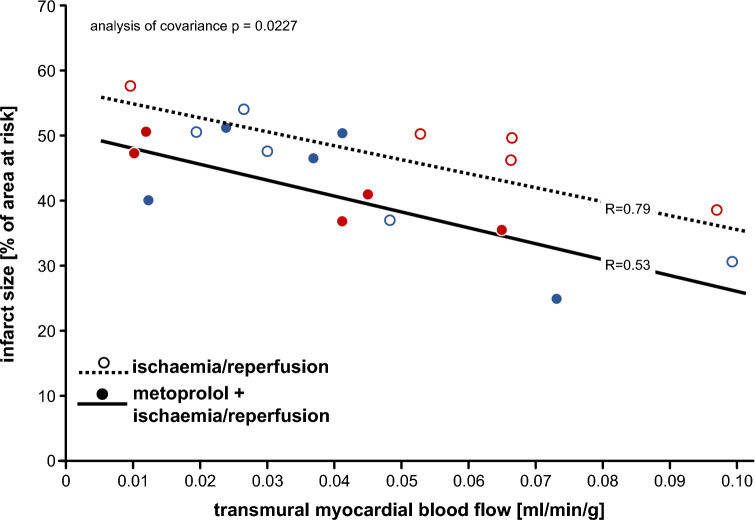
Relationships between infarct size as a fraction of area at risk and transmural blood flow to the ischaemic area at risk at 55-min ischaemia without (open circles) and with (closed circles) metoprolol in female (red) and male (blue) Göttingen minipigs. Linear regression lines were compared by analysis of covariance

**Fig. 3:**
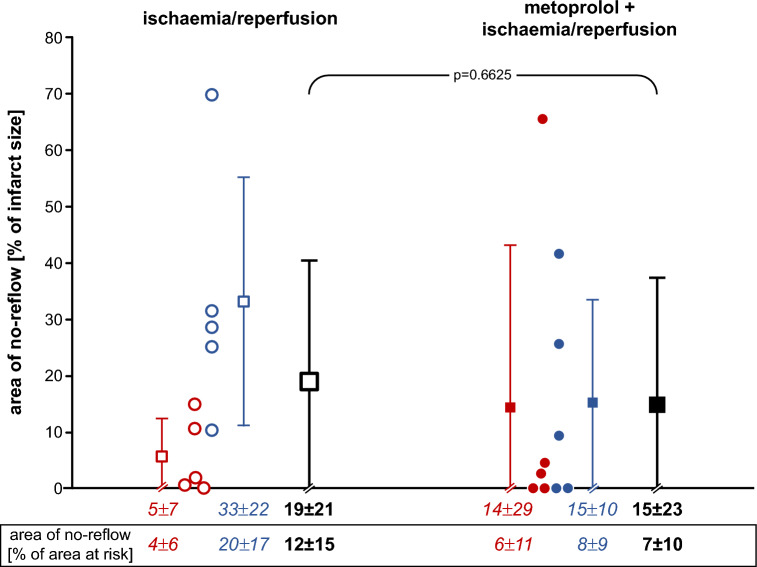
Area of no-reflow following ischaemia/reperfusion without (open symbols) and with 1.0 mg kg^−1^ metoprolol (filled symbols) in female (red) and male (blue) Göttingen minipigs; individual data (circles) and means (squares) with SD; means ± SD were also given as numerical data as a fraction of the infarct size and as a fraction of the area at risk (see the box below the x-axis; without versus with metoprolol: *p* = 0.368); females and males with ischaemia/reperfusion (*n* = 5, each); females and males with metoprolol + ischaemia/reperfusion (*n* = 5, each). Data were analysed by unpaired two-sided t-tests after aligned-rank transformation of the not normally distributed no-reflow data

Infarct size was not significantly different between pigs without and with 1 mg kg^−1^ metoprolol, neither in females (*p* = 0.1970) nor in males (*p* = 0.8634) (Fig. 1). Even with inclusion of the two above pigs with blood flow during ischaemia > 0.1 ml min^−1^ g^−1^, infarct size was not significantly reduced (*p* = 0.3017). The linear regressions between infarct size and transmural blood flow to the ischaemic area at risk per se were not strong due to the (per definition) limited flow range, and the regressions without and with metoprolol were only modesty different, but this difference was nevertheless significant (Fig. 2). Thus, for any given flow metoprolol reduced infarct size, whereas the flow data were shifted to lower values. The area of no-reflow was not different between pigs without and with 1 mg kg^−1^ metoprolol, neither in females (*p* = 0.5169) nor in males (*p* = 0.2018) (Fig. 3).

With twice 1 mg kg^−1^ (pretreatment plus treatment from 30- to 40-min ischaemia) in an additional 4 pigs, infarct size was also not reduced (metoprolol: 54 ± 9% of area at risk vs. contemporary placebo: 46 ± 8%, n.s.), but area of no-reflow tended to be increased when expressed as fraction of infarct size (metoprolol: 59 ± 20% of infarct size vs. contemporary placebo: 29 ± 12%, p = 0.0727) or as fraction of the area at risk (metoprolol: 37 ± 16% of area at risk vs. contemporary placebo: 15 ± 7%, p = 0.0873).

## Discussion

Contrary to our expectations, we did not see robust infarct size reduction by metoprolol in our translationally relevant pig model of reperfused acute myocardial infarction [39, 55, 57]. Neither infarct size, our primary endpoint (Fig. 1), nor coronary microvascular obstruction, a secondary endpoint, were reduced (Fig. 3). Thus our data differ from those of Ibanez and collaborators, [41, 43, 59] and this discrepancy reflects on the preclinical level the discrepancy seen on the clinical level between the METOCARD [42] and the EARLY-BAMI trials [68].

Of note, however, the inverse relationships between infarct size and regional myocardial blood flow in the area at risk reflected a modest direct infarct size reduction for any given blood flow by metoprolol, in association with a modest reduction in blood flow, most likely by unopposed alpha-adrenergic coronary constrictor tone. In fact, beta blockade could have unmasked alpha-adrenoceptor activation in the presence of the sympathetic activation of myocardial ischaemia/reperfusion [35]. Although alpha-adrenergic coronary vasoconstriction is less pronounced in pigs than in dogs or humans, [15, 72] it could nevertheless have contributed to impaired coronary microvascular blood flow with beta blockade [25] during ischaemia and reperfusion. Indeed, the metoprolol group had somewhat lower blood flow during ischaemia, but not during early reperfusion where, however, no-reflow areas with surrounding hyperaemic areas make blood flow highly heterogeneous and the measured blood flow is, by definition, not that in the no-reflow area. The opposing effects of direct protection and unopposed alpha-adrenergic coronary vasoconstriction resulted in no net change in infarct size, our primary endpoint. Unfortunately, Ibanez et al [41, 43, 59]. reported no data on ischaemic blood flow, but our infarct size vs. flow data may nevertheless mechanistically reconcile some of the discrepancy between our and their data on metoprolol´s effect on infarct size.

Why did we not see the significant infarct size reduction which Ibanez et al. had reported in their pig studies? There are a number of differences between our present study and the prior studies by Ibanez and colleagues [41, 43, 59] that deserve careful discussion and consideration (Table 3). The strain of pigs is an important variable in pig models of myocardial infarction, [76] and we used Göttingen minipigs as compared to Yorkshire [41, 43] or Large White pigs [59] used by Ibanez et al. In fact, we have recently shown that Ossabaw minipigs which have the genetic predisposition to develop a metabolic syndrome cannot be protected by local or remote ischaemic conditioning even before they develop the phenotype of the metabolic syndrome [50, 56]. Thus, a primordial non-responsiveness to infarct size reduction in pig strains indeed exists, [34] but there is no evidence for it in Göttingen minipigs [48, 49]. When considering a primordial non-responsiveness to cardioprotection with a translational perspective, [34] differences in the ethnic/genetic background of METOCARD and EARLY-BAMI could also to some extent account for their different outcome. The Göttingen minipigs in the present study definitely responded to metoprolol as expected for a beta blocker since haemodynamic responses to adrenaline were attenuated (Table 1).

**Table 3:**
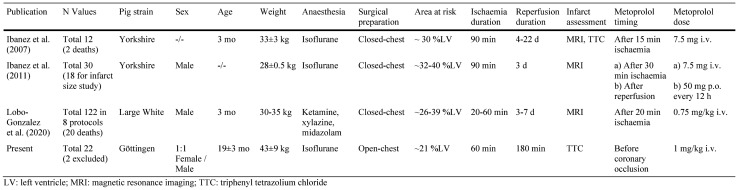
Differences between the studies from the Ibanez et al., Lobo-Gonzalez et al., and the present one on the effects of metoprolol on infarct size in pigs

Ibanez et al. in their original study did not report the sex of the Yorkshire pigs, [41, 43] and they used only male Large White pigs in their more recent and more comprehensive study. [59] Since the need to also consider for sex differences is emphasised in recent recommendations for preclinical [58] and clinical studies, [27] we used minipigs of both female and male sex. However, we have previously demonstrated that sex is not important for infarct size reduction by ischaemic preconditioning in Göttingen minipigs, [48] and therefore we believe, that sex does not account for the differences between our present and the prior Ibanez et al. studies.

The age of pigs differed substantially between our present study and those by Ibanez et al. Whereas their Yorkshire [41, 43] and Large White pigs [59] were only 3 months of age, corresponding to young children, we used adult sexually mature minipigs of 19 ± 2 months of age. With increasing age, the biological potential for protection and repair is diminished, [3, 8, 24] and acute myocardial infarction in humans occurs in patients of advanced age. Thus, age may indeed be an important variable to account for differences between our and the prior Ibanez et al. studies, and metoprolol may indeed exert greater cardioprotection at younger age.

Clearly, the anaesthetic regime is important for cardioprotection studies in pigs, [76] and volatile anaesthesia is more cardioprotective and may also facilitate cardioprotection more than intravenous anaesthesia e.g. with propofol in humans [52, 82]. We used an anaesthetic regime which is identical to that used in patients undergoing cardiovascular surgery in our institution [78] and uses continuous anaesthesia with isoflurane. So, isoflurane per se may have induced some cardioprotection in our studies and thus have limited the potential for further protection by metoprolol. Of note, however, Ibanez et al. in their original studies [41, 43] had also used continuous anaesthesia with isoflurane, and that did not prevent infarct size reduction by metoprolol, thus largely excluding the mode of anaesthesia as an important cause for differences between our and the Ibanez et al. studies. We acknowledge that patients with acute myocardial infarction and undergoing PCI are not anaesthetised, but that caveat pertains to both our and the Ibanez et al. studies.

The surgical preparation differed between our present and the Ibanez et al. studies. We used the open-chest preparation as used for the original infarct size reduction studies by Maroko, Ross and Braunwald et al [61]. and the original ischaemic conditioning studies by Murry, Reimer and Jennings [62]. We realise and admit that the open-chest preparation is more artificial than the closed-chest preparation used by Ibanez et al [41, 43, 59]. and that the trauma and open-chest condition per se may induce a pro-inflammatory condition. However, if indeed metoprolol exerts its protective effect through an anti-inflammatory action, our preparation should have been in a good position to evidence such anti-inflammatory action, but in fact did not.

The area at risk in our model was about 20% and, thus, smaller than in the model of Ibanez et al. with about 30% [41, 43, 59] and possibly also smaller than in the two clinical trials where the majority of patients had proximal LAD infarcts. However, with an infarct size of about 45% of the area at risk after 60-min ischaemia in the placebo group of our study, infarct progression was not complete and there was obviously sufficient room for infarct size reduction, as seen in our recent studies with ischaemic preconditioning where we reported an infarct size reduction by ischaemic preconditioning from about 45% to about 20–30% of the area at risk using our present model [48]

The duration of ischaemia is, of course, a major determinant of infarct size [33]. We used a standard ischaemia duration of 60 min which resulted in an infarct size of 46% of the area at risk in the placebo group. Ibanez et al. in their studies looked an infarct size with ischaemia durations between 30- and 90-min ischaemia and observed infarct sizes between 70 and 100% of the area at risk in their placebo groups; [41, 43, 59] the greatest reduction of infarct size with metoprolol was observed with 35-min ischaemia, where infarct size was reduced from about 80% to about 40% of the area at risk [59]. As outlined above, we have recently reported an infarct size reduction by ischaemic preconditioning from about 45% to about 20–30% of the area at risk using our present model, and there was obviously sufficient room for infarct size reduction with ischaemia duration of 60 min [48].

Definitely, as for all cardioprotective interventions reperfusion is a prerequisite for eventual infarct size reduction [29, 33] also for metoprolol, as it does not reduce infarct size in anaesthetised closed-chest pigs with permanent coronary occlusion [44]. In fact, as already acknowledged in the original report of Murry et al. for ischaemic preconditioning, [62] probably all cardioprotective interventions delivered before or during ischaemia just work to delay infarct progression and reduce infarct size only in conjunction with timely reperfusion [29]. The duration of reperfusion is of paramount importance for infarct size. The spatial development of infarct size over time is a complex process of distinct biological processes [31, 83]. The immediate phase of injury by ischaemia/reperfusion per se is followed after several hours by an inflammatory phase with a bimodal oedema development, [18] initial neutrophil [60] and subsequent macrophage infiltration [6] which serve to remove cellular debris and then initiate a healing response [16] of angiogenesis, fibroblast activation and resolution of inflammation over about 2 weeks, [19, 20] to then transition into a prolonged phase of remodelling which may be favourable and result in infarct (scar) size shrinkage or detrimental with infarct (scar) size expansion [38]. With 3-h reperfusion in our present study, we assessed probably only the infarct size which is caused by the immediate ischaemia/reperfusion injury. In a translational perspective, this immediate injury is probably most relevant for patients of higher Killip classes and their potential early in-hospital mortality; however, exactly those patients are in need of adjunct cardioprotection beyond that by reperfusion [36]. Infarct size as assessed by Ibanez et al. after 4–22 days reperfusion [41, 43, 59] comprises the acute injury plus the inflammatory/healing phase. Such infarct size is probably more relevant for the majority of patients who survive the early post-infarct phase. Still, infarct size after 22 days does not reflect the either favourable or detrimental remodelling process which may be most important for the development of heart failure and late mortality [23]. We, in our present study, could only assess the potential effect of metoprolol on the immediate ischaemia/reperfusion injury, whereas Ibanez et al. by assessing infarct size at between 4- and 22-day reperfusion also included the inflammatory/healing phase. Given that metoprolol exerts its potential beneficial effect through an anti-neutrophil action, [10, 41] it is obvious that such beneficial effect was seen in their studies but could not be seen in our study. The non-class effect of metoprolol in such beneficial action is still controversial [37]. Ibanez et al. previously reported that carvedilol attenuated macrophage infiltration much more than metoprolol in an anaesthetised pig model of 90-min coronary occlusion and 24-h reperfusion, [9] whereas they emphasised a non-class effect of metoprolol to attenuate neutrophil infiltration and reduce infarct size in their mouse model of 45-min coronary occlusion/ 24-h reperfusion [10].

The method of infarct size quantification–TTC in our present study vs. TTC and MRI [43] or MRI [59] in the Ibanez et al. studies–is probably not import for the discrepancy of data, since in their original study on infarct size reduction by metoprolol infarct size by MRI correlated very well with that by TTC [43].

The timing of metoprolol administration was different between our present and the Ibanez et al. studies. Whereas metoprolol was given intravenously 15 min [43], 20 min [59] or 30 min [41] after the start of the coronary occlusion in their studies, we used pretreatment immediately before coronary occlusion. We, thus, followed the advice of Ibanez et al. that, for effective cardioprotection, treatment with metoprolol should start as early as possible [41, 59]. Also, since Ibanez et al. proposed that metoprolol delays the progression of infarct development by about 15 min [59], our mode of administration should have done so, and there was sufficient duration of action as there was effective inhibition of peak haemodynamic responses to adrenaline 30–60 min after metoprolol administration (Table 1). A persistent action of metoprolol was also apparent from its attenuation of heart rate increase during reperfusion.

The dose of metoprolol is, of course, decisive for its efficacy. Metoprolol at the dose used in the present study attenuated the haemodynamic responses to adrenaline as expected for a beta blocker (Table 1). Somewhat surprisingly, metoprolol in our present study reduced heart rate only slightly as compared to the placebo group at some time points during the protocol, such that a reduction of ischaemic burden [25, 26] and infarct size [73] through the bradycardic action of beta blockade [28] was probably not recruited. Heart rate reduction by metoprolol was also not seen in the pig studies by Ibanez et al. which reported an infarct size reduction, [41, 43, 59] whereas some bradycardia was seen in both, the METOCARD and the EARLY-BAMI trials, however—as mentioned above—with equivocal effects on infarct size [42, 68]. As compared to a clinical setting, our total dose of about 40 mg intravenous metoprolol was certainly large, but still its haemodynamic effects were modest, suggesting potential species differences. To give metoprolol the best chance of exerting cardioprotection, we used an even somewhat higher dose than Ibanez et al. (1 mg kg^−1^ i.v. rather than 7.5 mg total dose [41, 43] or 0.75 mg kg^−^.^1^ i.v. [59]). ST-segment elevation was not attenuated by metoprolol, as would have been expected from an anti-ischaemic action during ongoing coronary occlusion [46, 70]. The number of episodes with ventricular fibrillation/defibrillation was not different between the metoprolol and the placebo group in our present study, in contrast to the prior studies of Ibanez et al. who, however, had used metoprolol and placebo on top of continuous amiodarone [43, 59] which we avoided because it reduces infarct size per se [13].

With our attempt to strengthen a protection by metoprolol by administration of a second 1 mg kg^−1^ i.v. dose at 30–40-min ischaemia, we also saw no infarct size reduction, further excluding insufficiency of the dose. At the very low regional myocardial blood flow during ischaemia, there may have been insufficient delivery of metoprolol to the ischaemia area. However, during reperfusion delivery of metoprolol was no longer limited, and we found even a trend for an increase in the no-reflow area above that in the placebo and the single dose metoprolol groups. Confirming a prior study on seasonal variation of no-reflow, [79] it was more marked in the 3 female contemporary placebo controls for the twice 1 mg kg^−1^ metoprolol pigs than in the placebo females for the 1 mg kg^−1^ metoprolol. With this second dose of metoprolol, substantial inhibition of beta adrenoceptors into the first 10 min of reperfusion was still present (Table 1). While with the single 1 mg kg^−^.^1^ i.v. dose pretreatment before coronary occlusion the action of metoprolol may have no longer been sufficient to attenuate the development of no-reflow at early reperfusion, the no-reflow area tended to be larger with the additional second dose of metoprolol at 30-min coronary occlusion than that with placebo or single dose, indicating that metoprolol in fact aggravated coronary microvascular injury. Again, beta blockade could have unmasked alpha-adrenoceptor activation also during reperfusion. Also, lack of beta adrenergic inhibition and enhanced alpha-adrenergic facilitation of platelet aggregation [2] could have contributed to the enhanced coronary microvascular obstruction [32, 63]. In our present study, therefore, the increased no-reflow at the higher metoprolol dose could be considered as an undesired side effect; as these additional data were underpowered, they can only serve to evidence lack of greater benefit from metoprolol at higher dose. We definitely did not find the attenuation of coronary microvascular obstruction suggested by Ibanez et al. from their studies in murine hearts where neutrophil function was attenuated by metoprolol [10, 21] and which were confirmed in a retrospective analysis of patients in the METOCARD trial [21].

Obviously, an array of subtle differences, such as genetic differences in the pig strains, [50] in age and sexual maturity, in anaesthesia, in experimental preparation (area at risk) and protocol (timing and dosing of metoprolol), and in quantification methods for infarct size and no-reflow area measurements, have obscured a potential cardioprotection by metoprolol in our present study, very similar as previously seen with remote ischaemic conditioning in rats of different strains and ages [54, 71]. Taking the above variables for potential differences between our and the Ibanez et al. studies into account, the age of pigs (adult, sexually mature vs. juvenile, immature) and the duration of reperfusion appear to be most important. The potential for protection and repair is diminished with increasing age, and patients with acute myocardial infarction are of advanced age. In a translational perspective, this is to the advantage of our present study. However, the duration of reperfusion is probably to the advantage of the Ibanez et al. in its translation to the majority of patients who have survived the immediate reperfusion phase over the first few days.

The lack of robustness of protection in our present study face-to-face with the prior studies by Ibanez et al. reflects the lack of robustness of infarct size reduction by metoprolol in patients with reperfused acute myocardial infarction seen in the equivocal METOCARD and EARLY-BAMI trials. Whereas we did not confirm cardioprotection by metoprolol in the acute phase of reperfused myocardial infarction for which the term “cardioprotection” is used *stricto *sensu and measured as infarct size reduction [55] the anti-neutrophil action of metoprolol nevertheless probably exerted protective effects during the healing and remodelling processes following reperfusion in the Ibanez et al. studies. They looked at infarct size in pigs by MRI after 3–7 [41, 43] or 7–45 days [22, 59] reperfusion, and they identified an anti-neutrophil action in mice after 6-h reperfusion [21], reduced neutrophil infiltration and infarct size after 24-h reperfusion in pigs, [41] and reduced infarct size and coronary microvascular obstruction in patients with acute myocardial infarction by MRI after 1-week reperfusion [21, 42], respectively. However, the EARLY-BAMI trial also quantified infarct size by MRI after 30-day reperfusion, when the healing phase was probably completed, and found no cardioprotection [68].

### Limitations

Unfortunately, we did not and could not mechanistically define the discrepancy between the Ibanez et al. and our data. To systematically resolve such discrepancy would require to exactly repeat the Ibanez et al. study and then compare the strains (Yorkshire, Large White vs. Göttingen) at both ages (immature vs. adult), at the two different anaesthetic regimes, in closed-chest vs. open-chest preparations, without and with use of anti-arrhythmics, with metoprolol early during vs. before ischaemia, at different durations of reperfusion (3 h vs. 5 days), with any combination of these conditions, and, of course, each of those comparisons with respective contemporary placebo controls, possibly also with a comparison of MRI vs. histology data on infarct size and no-reflow. Only after the condition which is possibly responsible for the discrepancy has been identified, would a mechanistic analysis of a difference in signal transduction make sense. With respect to the 3R of animal experimentation, [80] costs and efforts we considered this approach not justified. Our data just highlight the many experimental conditions which can impact on the result of a cardioprotection study and demonstrate that the lack of translation may not just be a disconnect between animals and humans. Similar differences in the ethnic background of patients and in local institutional practices, e.g. platelet inhibitor and heparin pretreatment, pain management, contrast agents for angiography, catheter and stent material, direct stenting vs. predilation, use of anti-arrhythmics and nitrates may exist in PCI of patients with acute myocardial infarction, impact on the result of a cardioprotective intervention and may have accounted for the differences between METOCARD and EARLY-BAMI. Therefore, given that the mechanistic cause for potentially discrepant results may remain unknown, it is even more important that the cardioprotective intervention under study is robust. This was not the case for infarct size reduction by metoprolol in our present study.

## Conclusion

**Fig. 4:**
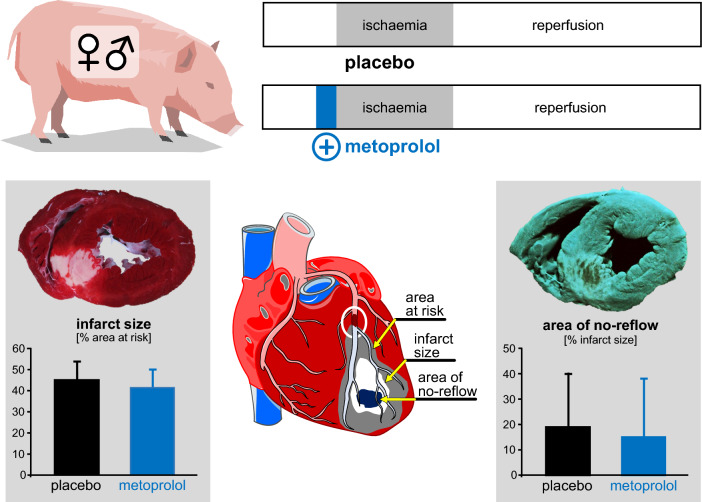
Schematic summary. Pretreatment with metoprolol before 60-min coronary occlusion and 180-min reperfusion did not reduce infarct size and coronary microvascular obstruction. Cardioprotection by metoprolol in pigs is not robust, and this result reflects the equivocal clinical trials

Different from prior studies by Ibanez et al. in a pig model of reperfused myocardial infarction, we found no infarct size reduction by metoprolol pretreatment in our open-chest Göttingen pig model of reperfused myocardial infarction (Fig. 4). The lack of protection may have resulted from opposite modest direct protection by metoprolol at any given blood flow along with a modest reduction of blood flow, possibly by alpha-adrenergic coronary vasoconstriction. Our data are not to be taken to refute the data of Ibanez et al. nor to mechanistically explain the different results, but they demonstrate that a number of details in a preclinical large animal model, such as strain, age and sexual maturity, anaesthesia, surgical preparation, protocol of ischaemia and reperfusion, dosing and timing of drug administration all add up to yield very different results with the same agent. All of these subtle differences will also occur in clinical trials and even more so in clinical practice. Infarct size reduction by metoprolol must, therefore, be considered as not robust, not only in clinical trials but also on the preclinical level in a pig model of reperfused acute myocardial infarction. These data reinforce the need for more rigorous preclinical studies in cardioprotection research before embarking on clinical trials, and the COST ACTION CARDIOPROTECTION consensus, therefore, advocates multi-centre studies in large animals, where each laboratory contributes by using its own standard procedures [5, 55].

### Supplementary Information

Below is the link to the electronic supplementary material.

Supplementary file1 (PDF 86 KB)

Supplementary file2 (PDF 89 KB)

## Data Availability

All supporting data of the present study are available in the article and its supplementary information.

## References

[1] Amanakis G, Kleinbongard P, Heusch G, Skyschally A (2019). Attenuation of ST-segment elevation after ischemic conditioning maneuvers reflects cardioprotection online. Basic Res Cardiol.

[2] Anfossi G, Trovati M (1996). Role of catecholamines in platelet function: pathophysiological and clinical significance. Eur J Clin Invest.

[3] Boengler K, Schulz R, Heusch G (2009). Loss of cardioprotection with ageing. Cardiovasc Res.

[4] Bolli R (2021). CAESAR's legacy: a new era of rigor in preclinical studies of cardioprotection. Basic Res Cardiol.

[5] Bolli R, Tang XL (2022). New insights into cardioprotection, gained by adopting the CAESAR standards of rigor. Basic Res Cardiol.

[6] Bönner F, Gastl M, Nienhaus F, Rothe M, Jahn A, Pfeiler S, Gross U, Schultheiss HP, Ibanez B, Kozerke S, Szendroedi J, Roden M, Westenfeld R, Schrader J, Flögel U, Heusch G, Kelm M (2022). Regional analysis of inflammation and contractile function in reperfused acute myocardial infarction by in vivo (19)F cardiovascular magnetic resonance in pigs. Basic Res Cardiol.

[7] Bøtker HE, Hausenloy D, Andreadou I, Antonucci S, Boengler K, Davidson SM, Deshwal S, Devaux Y, Di Lisa F, Di Sante M, Efentakis P, Femmino S, Garcia-Dorado D, Giricz Z, Ibanez B, Iliodromitis E, Kaludercic N, Kleinbongard P, Neuhauser M, Ovize M, Pagliaro P, Rahbek-Schmidt M, Ruiz-Meana M, Schlüter KD, Schulz R, Skyschally A, Wilder C, Yellon DM, Ferdinandy P, Heusch G (2018). Practical guidelines for rigor and reproducibility in preclinical and clinical studies on cardioprotection. Basic Res Cardiol.

[8] Broughton KM, Wang BJ, Firouzi F, Khalafalla F, Dimmeler S, Fernandez-Aviles F, Sussman MA (2018). Mechanisms of Cardiac Repair and Regeneration. Circ Res.

[9] Cimmino G, Ibanez B, Giannarelli C, Prat-Gonzalez S, Hutter R, Garcia M, Sanz J, Fuster V, Badimon JJ (2011). Carvedilol administration in acute myocardial infarction results in stronger inhibition of early markers of left ventricular remodeling than metoprolol. Int J Cardiol.

[10] Clemente-Moragón A, Gómez-Tech M, Villena-Gutierrez R, Lalama Tech DV, García-Prieto J, Martínez F, Sánchez-Cabo F, Fuster V, Oliver E, Ibánez B (2020). Metoprolol exerts a non-class effect against ischemia-reperfusion injury by abrogating exacerbated inflammation. Eur Heart J.

[11] Cooper HA, de Lemos JA, Morrow DA, Sabatine MS, Murphy SA, McCabe CH, Gibson CM, Antman EM, Braunwald E (2002). Minimal ST-segment deviation: a simple, noninvasive method for identifying patients with a patent infarction-related artery after fibrinolytic administration. Am Heart J.

[12] Curtis MJ, Alexander SPH, Cirino G, George CH, Kendall DA, Insel PA, Izzo AA, Ji Y, Panettieri RA, Patel HH, Sobey CG, Stanford SC, Stanley P, Stefanska B, Stephens GJ, Teixeira MM, Vergnolle N, Ahluwalia A (2022). Declaration of transparency and scientific rigour: Checklist for design and analysis 2022. Br J Pharmacol.

[13] DeBoer LW, Nosta JJ, Kloner RA, Braunwald E (1982). Studies of amiodarone during experimental myocardial infarction: beneficial effects on hemodynamics and infarct size. Circulation.

[14] Diaz-Munoz R, Valle-Caballero MJ, Sanchez-Gonzalez J, Pizarro G, Garcia-Rubira JC, Escalera N, Fuster V, Fernandez-Jimenez R, Ibanez B (2021). Intravenous metoprolol during ongoing STEMI ameliorates markers of ischemic injury: a METOCARD-CNIC trial electrocardiographic study. Basic Res Cardiol.

[15] Duncker DJ, Stubenitsky R, Verdouw PD (1998). Autonomic control of vasomotion in the porcine coronary circulation during treadmill exercise. Evidence for feed-forward ß-adrenergic control. Circ Res.

[16] Ertl G, Frantz S (2005). Wound model of myocardial infarction. Am J Physiol Heart Circ Physiol.

[17] Ferdinandy P, Andreadou I, Baxter GF, Bøtker HE, Davidson SM, Dobrev D, Gersh BJ, Heusch G, Lecour S, Ruiz-Meana M, Zuurbier CJ, Hausenloy DJ, Schulz R (2023). Interaction of cardiovascular nonmodifiable risk factors, comorbidities and comedications with ischemia/reperfusion injury and cardioprotection by pharmacological treatments and ischemic conditioning. Pharmacol Rev.

[18] Fernandez-Jimenez R, Barreiro-Perez M, Martin-Garcia A, Sanchez-Gonzalez J, Aguero J, Galan-Arriola C, Garcia-Prieto J, Diaz-Pelaez E, Vara P, Martinez I, Zamarro I, Garde B, Sanz J, Fuster V, Sanchez PL, Ibanez B (2017). Dynamic edematous response of the human heart to myocardial infarction: Implications for assessing myocardial area at risk and salvage. Circulation.

[19] Frangogiannis NG (2018). Cell biological mechanisms in regulation of the post-infarction inflammatory response. Curr Opin Physiol.

[20] Frangogiannis NG, Smith CW, Entman ML (2002). The inflammatory response in myocardial infarction. Cardiovasc Res.

[21] Garcia-Prieto J, Villena-Gutierrez R, Gomez M, Bernardo E, Pun-Garcia A, Garcia-Lunar I, Crainiciuc G, Fernandez-Jimenez R, Sreeramkumar V, Bourio-Martinez R, Garcia-Ruiz JM, Del Valle AS, Sanz-Rosa D, Pizarro G, Fernandez-Ortiz A, Hidalgo A, Fuster V, Ibanez B (2017). Neutrophil stunning by metoprolol reduces infarct size. Nat Commun.

[22] Garcia-Ruiz JM, Fernandez-Jimenez R, Garcia-Alvarez A, Pizarro G, Galan-Arriola C, Fernandez-Friera L, Mateos A, Nuno-Ayala M, Aguero J, Sanchez-Gonzalez J, Garcia-Prieto J, Lopez-Melgar B, Martinez-Tenorio P, Lopez-Martin GJ, Macias A, Perez-Asenjo B, Cabrera JA, Fernandez-Ortiz A, Fuster V, Ibanez B (2016). Impact of the timing of metoprolol administration during STEMI on infarct size and ventricular function. J Am Coll Cardiol.

[23] Gonzalez A, Ravassa S, Beaumont J, Lopez B, Diez J (2011). New targets to treat the structural remodeling of the myocardium. J Am Coll Cardiol.

[24] Gude NA, Broughton KM, Firouzi F, Sussman MA (2018). Cardiac ageing: extrinsic and intrinsic factors in cellular renewal and senescence. Nat Rev Cardiol.

[25] Guth BD, Heusch G, Seitelberger R, Ross J (1987). Mechanism of beneficial effect of beta-adrenergic blockade on exercise-induced myocardial ischemia in conscious dogs. Circ Res.

[26] Guth BD, Heusch G, Seitelberger R, Ross J (1987). Elimination of exercise-induced regional myocardial dysfunction by a bradycardic agent in dogs with chronic coronary stenosis. Circulation.

[27] Haider A, Bengs S, Luu J, Osto E, Siller-Matula JM, Muka T, Gebhard C (2020). Sex and gender in cardiovascular medicine: presentation and outcomes of acute coronary syndrome. Eur Heart J.

[28] Heusch G (2008). Heart rate in the pathophysiology of coronary blood flow and myocardial ischaemia: benefit from selective bradycardic agents. Br J Pharmacol.

[29] Heusch G (2013). Cardioprotection: chances and challenges of its translation to the clinic. Lancet.

[30] Heusch G (2017). Critical issues for the translation of cardioprotection. Circ Res.

[31] Heusch G (2018). Cardioprotection research must leave its comfort zone. Eur Heart J.

[32] Heusch G (2019). Coronary microvascular obstruction: the new frontier in cardioprotection. Basic Res Cardiol.

[33] Heusch G (2020). Myocardial ischaemia-reperfusion injury and cardioprotection in perspective. Nat Rev Cardiol.

[34] Heusch G, Bøtker EH, Ferdinandy P, Schulz R (2023). Primordial non-responsiveness – a novel obstacle to cardioprotection. Eur Heart J.

[35] Heusch G, Deussen A, Thämer V (1985). Cardiac sympathetic nerve activity and progressive vasoconstriction distal to coronary stenoses: feed-back aggravation of myocardial ischemia. J Auton Nerv Syst.

[36] Heusch G, Gersh BJ (2020). Is cardioprotection salvageable?. Circulation.

[37] Heusch G, Kleinbongard P (2020). Is metoprolol more cardioprotective than other beta-blockers?. Eur Heart J.

[38] Heusch G, Libby P, Gersh B, Yellon D, Böhm M, Lopaschuk G, Opie L (2014). Cardiovascular remodeling in coronary artery disease and heart failure. Lancet.

[39] Heusch G, Skyschally A, Schulz R (2011). The in-situ pig heart with regional ischemia/reperfusion - ready for translation. J Mol Cell Cardiol.

[40] Homeister JW, Hoff PT, Fletcher DD, Lucchesi BR (1990). Combined adenosine and lidocaine administration limits myocardial reperfusion injury. Circulation.

[41] Ibanez B, Cimmino G, Prat-Gonzalez S, Vilahur G, Hutter R, Garcia MJ, Fuster V, Sanz J, Badimon L, Badimon JJ (2011). The cardioprotection granted by metoprolol is restricted to its administration prior to coronary reperfusion. Int J Cardiol.

[42] Ibanez B, Macaya C, Sanchez-Brunete V, Pizarro G, Fernandez-Friera L, Mateos A, Fernandez-Ortiz A, Garcia-Ruiz JM, Garcia-Alvarez A, Iniguez A, Jimenez-Borreguero J, Lopez-Romero P, Fernandez-Jimenez R, Goicolea J, Ruiz-Mateos B, Bastante T, Arias M, Iglesias-Vazquez JA, Rodriguez MD, Escalera N, Acebal C, Cabrera JA, Valenciano J, de Perez PA, Fernandez-Campos MJ, Casado I, Garcia-Rubira JC, Garcia-Prieto J, Sanz-Rosa D, Cuellas C, Hernandez-Antolin R, Albarran A, Fernandez-Vazquez F (2013). Effect of early metoprolol on infarct size in ST-segment-elevation myocardial infarction patients undergoing primary percutaneous coronary intervention: The effect of metoprolol in cardioprotection during an acute myocardial infarction (METOCARD-CNIC) trial. Circulation.

[43] Ibanez B, Prat-Gonzalez S, Speidl WS, Vilahur G, Pinero A, Cimmino G, Garcia MJ, Fuster V, Sanz J, Badimon JJ (2007). Early metoprolol administration before coronary reperfusion results in increased myocardial salvage: analysis of ischemic myocardium at risk using cardiac magnetic resonance. Circulation.

[44] Kern KB, Hilwig RW, Warner A, Basnight M, Ewy GA (1995). Failure of intravenous metropolol to limit acute myocardial infarct size in a nonreperfused porcine model. Am Heart J.

[45] Kleinbongard P (2020). Cardioprotection by early metoprolol attenuation of ischemic vs reperfusion injury?. Basic Res Cardiol.

[46] Kleinbongard P, Amanakis G, Skyschally A, Heusch G (2018). Reflection of cardioprotection by remote ischemic perconditioning in attenuated ST-segment elevation during ongoing coronary occlusion in pigs: Evidence for cardioprotection from ischemic injury. Circ Res.

[47] Kleinbongard P, Bøtker HE, Ovize M, Hausenloy DJ, Heusch G (2020). Co-morbidities and co-medications as confounders of cardioprotection - does it matter in the clinical setting?. Br J Pharmacol.

[48] Kleinbongard P, Lieder H, Skyschally A, Heusch G (2023). No sex-related differences in infarct size, no-reflow and protection by ischaemic preconditioning in Göttingen minipigs. Cardiovasc Res.

[49] Kleinbongard P, Lieder H, Skyschally A, Heusch G (2023). Diazoxide is a powerful cardioprotectant but not feasible in a realistic infarct scenario. Front Cardiovasc Med.

[50] Kleinbongard P, Lieder HR, Skyschally A, Alloosh M, Gödecke A, Rahmann S, Sturek M, Heusch G (2022). Non-responsiveness to cardioprotection by ischaemic preconditioning in ossabaw minipigs with genetic predisposition to, but without the phenotype of the metabolic syndrome. Basic Res Cardiol.

[51] Kloner RA, Das S, Poole K, Perrit R, Muller J, Cannon CP, Braunwald E (2001). Seasonal variation of myocardial infarct size. Am J Cardiol.

[52] Kottenberg E, Thielmann M, Bergmann L, Heine T, Jakob H, Heusch G, Peters J (2012). Protection by remote ischaemic preconditioning during coronary artery bypass grafting with isoflurane but not with propofol anesthesia - a clinical trial. Acta Anaesthesiol Scand.

[53] Kowallik P, Schulz R, Guth BD, Schade A, Paffhausen W, Gross R, Heusch G (1991). Measurement of regional myocardial blood flow with multiple colored microspheres. Circulation.

[54] Lassen TR, Hjortbak MV, Hauerslev M, Tonnesen PT, Kristiansen SB, Jensen RV, Botker HE (2021). Influence of strain, age, origin, and anesthesia on the cardioprotective efficacy by local and remote ischemic conditioning in an ex vivo rat model. Physiol Rep.

[55] Lecour S, Andreadou I, Bøtker HE, Davidson SM, Heusch G, Ruiz-Meana M, Schulz R, Zuurbier CJ, Ferdinandy P, Hausenloy DJ (2021). IMproving preclinical assessment of cardioprotective therapies (IMPACT) criteria: guidelines of the EU-Cardioprotection cost action. Basic Res Cardiol.

[56] Lieder HR, Skyschally A, Sturek M, Heusch G, Kleinbongard P (2022). Remote ischemic conditioning in Ossabaw minipigs induces the release of humoral cardioprotective triggers, but the myocardium does not respond with reduced infarct size. Am J Physiol Heart Circ Physiol.

[57] Lindsey ML, Bolli R, Canty JM, Du XJ, Frangogiannis NG, Frantz S, Gourdie RG, Holmes JW, Jones SP, Kloner R, Lefer DJ, Liao R, Murphy E, Ping P, Przyklenk K, Recchia FA, Schwartz Longacre L, Ripplinger CM, Van Eyk JE, Heusch G (2018). Guidelines for experimental models of myocardial ischemia and infarction. Am J Physiol Heart Circ Physiol.

[58] Lindsey ML, LeBlanc AJ, Ripplinger CM, Carter JR, Kirk JA, Hansell Keehan K, Brunt KR, Kleinbongard P, Kassiri Z (2021). Reinforcing rigor and reproducibility expectations for use of sex and gender in cardiovascular research. Am J Physiol Heart Circ Physiol.

[59] Lobo-Gonzalez M, Galan-Arriola C, Rossello X, Gonzalez-Del-Hoyo M, Vilchez JP, Higuero-Verdejo MI, Garcia-Ruiz JM, Lopez-Martin GJ, Sanchez-Gonzalez J, Oliver E, Pizarro G, Fuster V, Ibanez B (2020). Metoprolol blunts the time-dependent progression of infarct size. Basic Res Cardiol.

[60] Ma Y, Yabluchanskiy A, Iyer RP, Cannon PL, Flynn ER, Jung M, Henry J, Cates CA, Deleon-Pennell KY, Lindsey ML (2016). Temporal neutrophil polarization following myocardial infarction. Cardiovasc Res.

[61] Maroko PR, Kjekshus JK, Sobel BE, Watanabe T, Covell JW, Ross J, Braunwald E (1971). Factors influencing infarct size following experimental coronary artery occlusions. Circulation.

[62] Murry CE, Jennings RB, Reimer KA (1986). Preconditioning with ischemia: a delay of lethal cell injury in ischemic myocardium. Circulation.

[63] Niccoli G, Montone R, Ibanez B, Thiele H, Crea F, Heusch G, Bulluck H, Hausenloy D, Berry C, Stiermaier T, Camici P, Eitel I (2019). Optimized treatment of ST-elevation myocardial infarction: The unmet need to target coronary microvascular obstruction as primary treatment goal to further improve prognosis. Circ Res.

[64] Percie du Sert N, Ahluwalia A, Alam S, Avey MT, Baker M, Browne WJ, Clark A, Cuthill IC, Dirnagl U, Emerson M, Garner P, Holgate ST, Howells DW, Hurst V, Karp NA, Lazic SE, Lidster K, MacCallum CJ, Macleod M, Pearl EJ, Petersen OH, Rawle F, Reynolds P, Rooney K, Sena ES, Silberberg SD, Steckler T, Wurbel H (2020). Reporting animal research: explanation and elaboration for the ARRIVE guidelines. PLoS Biol.

[65] Percie du Sert N, Hurst V, Ahluwalia A, Alam S, Avey MT, Baker M, Browne WJ, Clark A, Cuthill IC, Dirnagl U, Emerson M, Garner P, Holgate ST, Howells DW, Karp NA, Lazic SE, Lidster K, MacCallum CJ, Macleod M, Pearl EJ, Petersen OH, Rawle F, Reynolds P, Rooney K, Sena ES, Silberberg SD, Steckler T, Wurbel H (2020). The Arrive guidelines 20: Updated guidelines for reporting animal research. PLoS Biol.

[66] Pizarro G, Fernandez-Friera L, Fuster V, Fernandez-Jimenez R, Garcia-Ruiz JM, Garcia-Alvarez A, Mateos A, Barreiro MV, Escalera N, Rodriguez MD, De MA, Garcia-Lunar I, Parra-Fuertes JJ, Sanchez-Gonzalez J, Pardillos L, Nieto B, Jimenez A, Abejon R, Bastante T, De V, Cabrera JA, Lopez-Melgar B, Guzman G, Garcia-Prieto J, Mirelis JG, Zamorano JL, Albarran A, Goicolea J, Escaned J, Pocock S, Iniguez A, Fernandez-Ortiz A, Sanchez-Brunete V, Macaya C, Ibanez B (2014). Long term benefit of early pre-reperfusion metoprolol administration in patients with acute myocardial infarction: results from the METOCARD-CNIC trial. J Am Coll Cardiol.

[67] Podlesnikar T, Pizarro G, Fernandez-Jimenez R, Montero-Cabezas JM, Sanchez-Gonzalez J, Bucciarelli-Ducci C, Ajmone Marsan N, Fras Z, Bax JJ, Fuster V, Ibanez B, Delgado V (2020). Five-year outcomes and prognostic value of feature-tracking cardiovascular magnetic resonance in patients receiving early prereperfusion metoprolol in acute myocardial infarction. Am J Cardiol.

[68] Roolvink V, Ibanez B, Ottervanger JP, Pizarro G, van Royen N, Mateos A, Dambrink JHE, Escalera N, Lipsic E, Albarran A, Fernández-Ortiz A, Fernández-Avilés F, Goicolea J, Botas J, Remkes W, Hernandez-Jaras V, Kedhi E, Zamorano JL, Navarro F, Alfonso F, García-Lledó A, Alonso J, van Leeuwen M, Nijveldt R, Postma S, Kolkman E, Gosselink M, de Smet B, Rasoul S, Piek JJ, Fuster V (2016). Early administration of intravenous beta blockers in patients with ST-elevation myocardial infarction before primary PCI. J Am Coll Cardiol.

[69] Roolvink V, Ottervanger JP, Ibanez B, Dambrink JH, Gosselink M, Kedhi E, van Royen N, Lipsic E, Remkes W, Piek J, Fuster V, van 't Hof A,  (2018). One-year clinical outcome of early administration of intravenous beta-blockers in patients with ST-segment elevation myocardial infarction before primary percutaneous coronary reperfusion. EuroIntervention.

[70] Rossello X, Ibanez B (2018). Infarct size reduction by targeting ischemic injury: Back to square one. Circ Res.

[71] Sayour NV, Brenner GB, Makkos A, Kiss B, Kovácsházi C, Gergely TG, Aukrust SG, Tian H, Zenkl V, Gömöri K, Szabados T, Bencsik P, Heinen A, Schulz R, Baxter GF, Zuurbier CJ, Vokó Z, Ferdinandy P, Giricz Z (2023). Cardioprotective efficacy of limb remote ischemic preconditioning in rats: discrepancy between meta-analysis and a three-centre in vivo study. Cardiovasc Res.

[72] Schulz R, Oudiz RJ, Guth BD, Heusch G (1990). Minimal a1- and a2-adrenoceptor-mediated coronary vasoconstriction in the anaesthetized swine. Naunyn Schmiedebergs Arch Pharmacol.

[73] Schulz R, Rose J, Skyschally A, Heusch G (1995). Bradycardic agent UL-FS 49 attenuates ischemic regional dysfunction and reduces infarct size in swine: comparison with the beta-blocker atenolol. J Cardiovasc Pharmacol.

[74] Skyschally A, Amanakis G, Neuhäuser M, Kleinbongard P, Heusch G (2017). Impact of electrical defibrillation on infarct size and no-reflow in pigs subjected to myocardial ischemia-reperfusion without and with ischemic conditioning. Am J Physiol Heart Circ Physiol.

[75] Skyschally A, Heusch G (2011). Reduction of myocardial infarct size by dronedarone in pigs-a pleiotropic action?. Cardiovasc Drugs Ther.

[76] Solanes N, Bobi J, Arrieta M, Jimenez FR, Palacios C, Rodriguez JJ, Roque M, Galan-Arriola C, Ibanez B, Freixa X, Garcia-Alvarez A, Sabate M, Rigol M (2022). An open secret in porcine acute myocardial infarction models: The relevance of anaesthetic regime and breed in ischaemic outcomes. Front Vet Sci..

[77] Te Lintel HM, Newton G, Chapman K, Aqil R, Downham R, Yan R, Merkus D, Whitlock G, Lane CAL, Cawkill D, Perrior T, Duncker DJ, Schneider MD (2021). Preclinical trial of a MAP4K4 inhibitor to reduce infarct size in the pig: does cardioprotection in human stem cell-derived myocytes predict success in large mammals?. Basic Res Cardiol.

[78] Thielmann M, Kottenberg E, Kleinbongard P, Wendt D, Gedik N, Pasa S, Price V, Tsagakis K, Neuhäuser M, Peters J, Jakob H, Heusch G (2013). Cardioprotective and prognostic effects of remote ischaemic preconditioning in patients undergoing coronary artery bypass surgery: a single-centre randomised, double-blind, controlled trial. Lancet.

[79] Uitterdijk A, Yetgin T, Te Lintel HM, Sneep S, Krabbendam-Peters I, van Beusekom HM, Fischer TM, Cornelussen RN, Manintveld OC, Merkus D, Duncker DJ (2015). Vagal nerve stimulation started just prior to reperfusion limits infarct size and no-reflow. Basic Res Cardiol.

[80] van der Velden J, Asselbergs FW, Bakkers J, Batkai S, Bertrand L, Bezzina CR, Bot I, Brundel B, Carrier L, Chamuleau S, Ciccarelli M, Dawson D, Davidson SM, Dendorfer A, Duncker DJ, Eschenhagen T, Fabritz L, Falcao-Pires I, Ferdinandy P, Giacca M, Girao H, Gollmann-Tepekoylu C, Gyongyosi M, Guzik TJ, Hamdani N, Heymans S, Hilfiker A, Hilfiker-Kleiner D, Hoekstra AG, Hulot JS, Kuster DWD, van Laake LW, Lecour S, Leiner T, Linke WA, Lumens J, Lutgens E, Madonna R, Maegdefessel L, Mayr M, van der Meer P, Passier R, Perbellini F, Perrino C, Pesce M, Priori S, Remme CA, Rosenhahn B, Schotten U, Schulz R, Sipido KR, Sluijter JPG, van Steenbeek F, Steffens S, Terracciano CM, Tocchetti CG, Vlasman P, Yeung KK, Zacchigna S, Zwaagman D, Thum T (2022). Animal models and animal-free innovations for cardiovascular research: current status and routes to be explored. Consensus document of the ESC working group on myocardial function and the ESC working group on cellular biology of the heart. Cardiovasc Res.

[81] Vilahur G (2023). A primordial obstacle in cardioprotection: the answer resides in our genes. Cardiovasc Res.

[82] Zangrillo A, Musu M, Greco T, Di Prima AL, Matteazzi A, Testa V, Nardelli P, Febres D, Monaco F, Calabro MG, Ma J, Finco G, Landoni G (2015). Additive effect on survival of anaesthetic cardiac protection and remote ischemic preconditioning in cardiac surgery: a Bayesian network meta-analysis of randomized trials. PLoS ONE.

[83] Zhang RYK, Cochran BJ, Thomas SR, Rye KA (2023). Impact of reperfusion on temporal immune cell dynamics after myocardial infarction. J Am Heart Assoc.

