# Supplementary material for: No robust reduction of infarct size and no-reflow by metoprolol pretreatment in adult Göttingen minipigs
Source: Basic Res Cardiol. 2023 Jun 8;118(1):23. doi: 10.1007/s00395-023-00993-4 (PMC10250284; doi:10.1007/s00395-023-00993-4)
Supplement: Supplementary file 1 — Supplementary file1 (PDF 86 KB) [file 395_2023_993_MOESM1_ESM.pdf]

Supplemental Table. Area at risk, regional myocardial blood flow, heart rate and left ventricular pressure in pigs undergoing ischaemia/reperfusion without and with twice 1 mg kg^{-1} (pretreatment plus treatment from 30 to 40 min ischaemia) metoprolol.

		I/R	metoprolol+I/R	statistics
**AAR (% LV)**		24±7	22±4	*p=0.5691*
**episodes of ventricular fibrillation/defibrillation**		2±2	2±1	*p=0.9321*
**regional myocardial blood flow (ml min^{-1}g^{-1})**	baseline	0.965± 0.151	0.856± 0.230	*protocol: p=0.5467; time: p<0.0001; time*protocol: p=0.5844*
	metoprolol		0.955± 0.234	
	I5	0.074± 0.021 *<0.0001 vs. baseline*	0.035± 0.014 *<0.0001 vs. baseline*	
	I55	0.038± 0.013 *<0.0001 vs. baseline*	0.021± 0.016 *<0.0001 vs. baseline*	
	R10	1.022± 0.217	0.663± 0.117 *0.0122 vs. baseline*	
	R180	0.780± 0.081 *0.0108 vs. baseline*	0.619± 0.165 *0.0021 vs. baseline*	
**heart rate (1 min^{-1})**	baseline	100± 0	99± 10	*protocol: p=0.2930; time: p=0.4032; time*protocol: p=0.4979*
	metoprolol		97± 7	
	I5	101± 1	97± 9	
	I55	96± 9	102± 3	
	R10	100± 4	104± 6	
	R180	102± 4	107± 13	
**LVP_{max} (mmHg)**	baseline	85± 2	96± 7	*protocol: p=0.0217; time: p<0.0001; time*protocol: p=0.5613*
	metoprolol		89± 10	
	I5	77± 6	77± 10 *<0.0001 vs. baseline*	
	I55	70± 10 *0.0004 vs. baseline*	69± 10 *<0.0001 vs. baseline*	
	R10	68± 9 *<0.0001 vs. baseline*	73± 12 *<0.0001 vs. baseline*	
	R180	65± 9 *<0.0001 vs. baseline*	73± 11 *0.0002 vs. baseline*	

Area at risk (AAR); ischaemia/reperfusion (I/R); 5 min ischaemia (I5); 55 min ischaemia (I55); left ventricle (LV); left ventricular pressure (LVP); 10 min reperfusion (R10); 180 min reperfusion (R180); area at risk was analysed by an unpaired two-sided t-test; the number of episodes of ventricular fibrillation/defibrillation during ischaemia was not normally distributed and subjected to an aligned-rank transformation (art) before analysis; haemodynamic and regional myocardial blood flow data were analysed by two-way analysis of variance (protocol. time) for repeated measures.
