# Supplementary material for: No robust reduction of infarct size and no-reflow by metoprolol pretreatment in adult Göttingen minipigs
Source: Basic Res Cardiol. 2023 Jun 8;118(1):23. doi: 10.1007/s00395-023-00993-4 (PMC10250284; doi:10.1007/s00395-023-00993-4)
Supplement: Supplementary file 2 — Supplementary file2 (PDF 89 KB) [file 395_2023_993_MOESM2_ESM.pdf]

Supplemental Figure

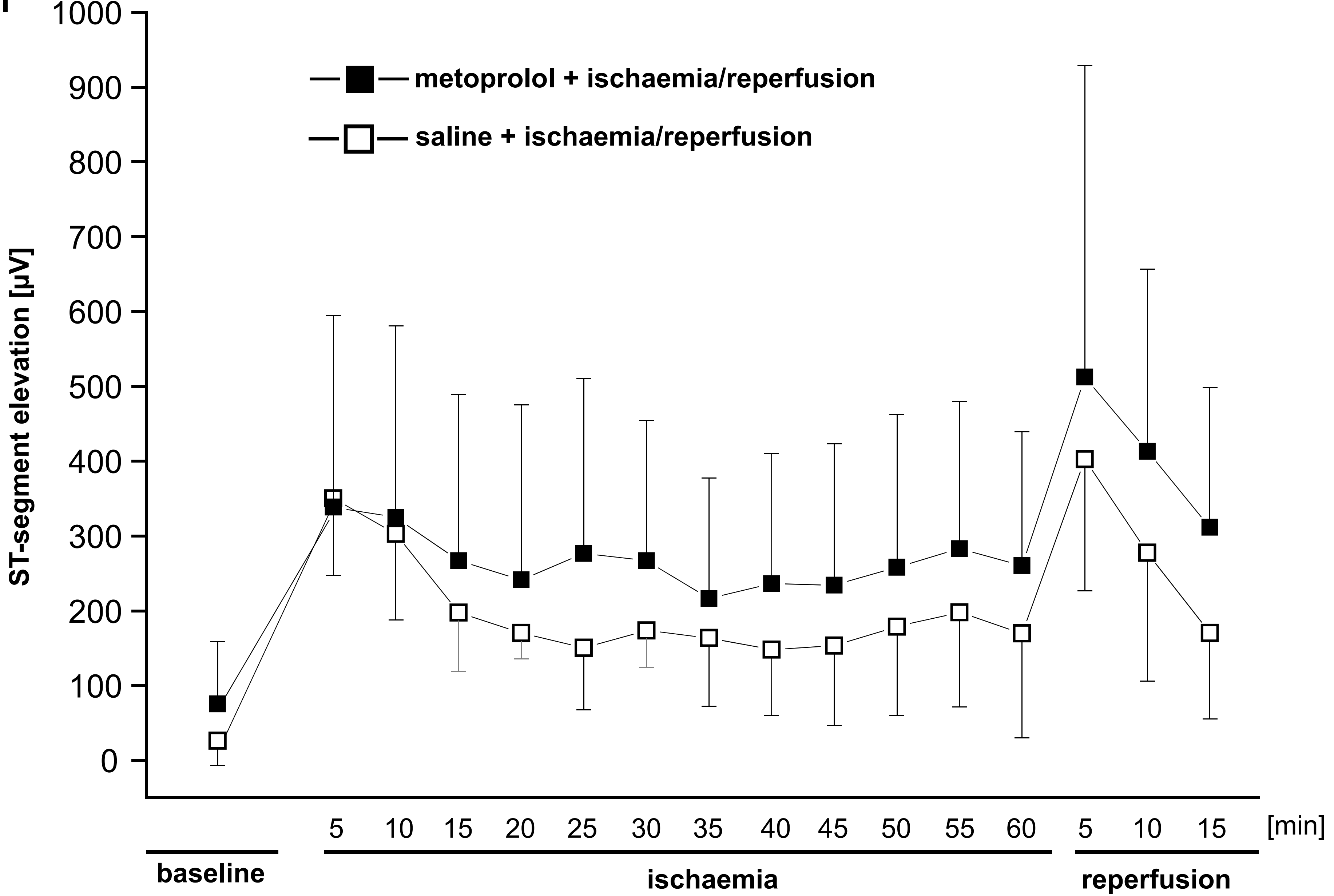

Time courses of ST-segment elevations from pigs treated with saline or metoprolol infusion (n=10, each) before 60 min coronary occlusion and 180 min reperfusion. Values for baseline were calculated from 60 consecutive cardiac cycles at 5-10 min of saline/metoprolol infusion. For all other timepoints averaged values of 30 consecutive cardiac cycles were calculated. Values are means ± standard deviations. Please note, that the ECG was recorded in a non-standardised fashion from an open-chest preparation just to online monitor heart rate and arrhythmias. Therefore, some data are missing, in detail the following measurements were excluded from the analysis in the saline + ischaemia/reperfusion group: during ischaemia: n=3 at 15 min, n=3 at 20 min, n=2 at 25 min, n=4 at 30 min, n=3 at 35 min, and during reperfusion: n=1 at 10 min. The following measurements were excluded from the analysis in the metoprolol + ischaemia/reperfusion group: during ischemia: n=1 at 20 min, n=2 at 25 min, n=2 at 30 min, n=2 at 35 min. The available date have a high variability. The data for the presented analysis were thus normalised for baseline before comparing the saline and metoprolol groups. Two-way analysis of variance for repeated measurements: group p=0.1669; time p<0.0001, time*group p=0.9962.
